# Cohort profile: Singapore’s nationally representative Retirement and Health Study with 5 waves over 10 years

**DOI:** 10.4178/epih.e2022030

**Published:** 2022-02-21

**Authors:** Reuben Ng, Yi Wen Tan, Kelvin Bryan Tan

**Affiliations:** 1 Lee Kuan Yew School of Public Policy, National University of Singapore, Singapore; 2 Lloyd’s Register Foundation Institute for the Public Understanding of Risk, National University of Singapore, Singapore; 3 Ministry of Health, Singapore

**Keywords:** Retirement, Health, Well-being, Employment, Longitudinal studies, Geriatrics

## Abstract

The Retirement and Health Study (RHS) is Singapore’s largest nationally representative cohort with over 15,000 participants (aged 45-85 years) followed across five timepoints in 10 years (2014-2024). Accounting for sample weights, the sample represents 1.2 million Singaporeans and permanent residents of a total population of 5.5 million. The RHS sought consent to link survey responses to relevant administrative data, enabling the cross-validation of self-reports with national databases. There are 10 sections in the RHS with over 400 questions, 50% of which are on respondents’ physical and mental health, healthcare utilization and insurance; the remaining 50% are about employment history, retirement adequacy, wealth, and household expenditure. The RHS was set up to provide microdata to compliment administrative data for whole-of-government policy making given that Singapore will reach super-aged status by 2026. Sample findings include a need for older adults to balance between immediate financial needs and investments regarding their pension funds. Also, 86% of older adults preferred to transit into partial retirement by reducing workloads. On the health front, existing studies utilising the RHS have revealed latent classes of disabilities, and that intentions to seek employment can mitigate disability developments. Another study reported that physical disability and social isolation was projected to increase, with ethnic disparities in social functioning. Overall, the RHS will be used for evidenced-informed policy agenda setting and evaluation across domains of health, finance, retirement adequacy, social and family development.

## INTRODUCTION

Retirement is a multifaceted decision with broad economic and social implications for modern society. Leaving the workforce has an inexplicable relationship with health outcomes and subsequent public policies [1,2]. However, the cost and benefits of retirement on health and wellbeing has seen conflicting results [1,3-5]. Some reviews find that retirement decreases stress, improve health perceptions, and lower the severity of medical diseases [1,6]. Other studies however found that retirement increase social isolation [7], and even advocate the benefits of working beyond retirement [8]. Evidently, different phases of retirement (i.e., early vs. late retirement, etc.) have unique effects on different dimensions of health (i.e., mental health, mortality, frailty, etc.) [6]. Demographic factors like age, gender, education, marital status and socioeconomic status (SES) moderate the relationship between retirement and health [1,6]. There is also little consensus regarding the cost and benefits of retirement on health across different countries [9]. These suggests that there is substantial heterogeneity of health experiences among retirees, making the relationship unique to each society.

Several Asian countries have conducted longitudinal aging and retirement studies [10], but none exists in Singapore [11,12]. Singapore has experienced a significant decline in birth rate since the early 1970s with historic lows averaging 1.19 over the past ten years [13,14]. In 2018, older adults aged 65 years and above equaled that of youths 15 years and below, described as a “demographic time bomb” [13]. By 2030, youths 15 years and below will plunge to 11% while older adults 65 years and older will reach 27% of the population [13]. Simultaneously, disability prevalence were projected to grow by five-fold in 40 years [15]. Medical ailments like, cardiovascular diseases (14.2% of total disability-adjusted life years), cancers (13.3%), musculoskeletal disorders (12.6%), mental disorders (10.2%) will be the top leading causes of disability-adjusted life years from 2017 in Singapore [13]. As a result, lifetime hospital expenditure is projected to increase by 30%, posing economic and policy difficulties for long term care [16]. Based on these predictions, trajectories and profiles of retirement and health are likely to diversity [17], presenting a challenge for policy makers on issues such as retirement adequacy, healthcare spending, long-term care, and psychosocial issues such as ageism [18-24]. Subsequently local policies need accurate data to format appropriate long-term health plans for the population.

To circumvent challenges related to the rise of aging in Singapore, the government convened an ‘Inter-ministerial Committee on the Ageing Population’ in 1999 to produce a report on the challenges, opportunities, and a policy roadmap to prepare for a rapidly aging population [25]. The report underscored the need for nationally representative cohort studies to inform policy [25], resulting in the Retirement and Health Study (RHS), Singapore’s largest longitudinal study of Singaporeans aged 45 years to 85 years with committed funding for five waves over 10 years (2014-2024). Collaboration between several agencies was undertaken to achieve a broader understanding of aging in Singapore, capturing several socioeconomic and wellbeing facets of aging. Government ministries include the Housing and Development Board (HDB; the agency that looks after the building and sales transactions of public housing where over 80% of the population reside), Ministry of Finance, Ministry of Health (MOH) and Ministry of Manpower —and is led by the Central Provident Fund Board, Singapore’s national pension fund administrator.

There are several purposes of the survey, one of which addresses the paucity of longitudinal studies with nationally representative cohorts in Singapore [26]. The RHS was designed to obtain information with a clear understanding of aging in Singapore, especially to map longitudinal trajectories of health, social, and economic developments of the aged population. This contribution provides microdata for policy making long-range planning for aging, and simultaneously informs research practices about the current state of health adequacy among older Singaporeans. Its aims are not restricted for local use and is aligned with international efforts to reframe aging [27-31], and preventive health policy developments especially during coronavirus disease 2019 [32-36]. Recent efforts in Southeast Asia have amassed several longitudinal surveys of the aged population across the region [37]. The RHS compliments these international efforts to enable comparative studies of aging across datasets of other countries. This RHS also contributes to the literature extant about the relationship between retirement and wellbeing, detailing trajectories of retirement, while accounting for a broad range of pertinent demographic factors, and its impact on several health outcomes.

## DATA SOURCE

### Study participants

In consultation with the Department of Statistics, we identified all households with members above 45 years of age that included Singaporean citizens, and permanent residents. Stratified random sampling was done based on age, gender, race and other demographic attributes based on the latest census of Singapore. We planned for a sample of 12,500, and with an expected response rate of 50%, our initial outreach went to 25,000 households. The RHS received a 60.4% (n= 15,013) response rate for the first wave. Data collection occurs every two years, with the first wave of data collected in 2014. Participants who consent were recontacted and invited to partake in subsequent waves. At present, data are available for the second wave conducted in 2016.

Proxy respondents were invited if they were above 21 years of age (i.e., spouse or children), and consent to represent participants who were unable to attend interviews due to physical or mental disability. Proxy respondents consisted of proximately 2.1% (n= 314) of the first wave, and approximately 1.2% (n= 151) of the second wave (Table 1). A separate consent form was approved by a legally acceptable representative (LAR) if the participant was unable to provide consent due to physical or mental disabilities, and approximately 0.8% (n= 120) of wave one, and 0.6% (n= 73) of wave two required an LAR (Table 1).

### Ethics statement

Ethics approval for the RHS was granted by the Health Promotion Board Medical and Dental Board (HP24:03/31-2).

### Attrition

Attrition is of concern for longitudinal studies, the RHS circumvents this in two ways. The RHS oversampled during the initial phase to maximize sample size to ensure a reasonable number of follow-ups. By the second wave 1.8% (n = 270) participants were deceased, an additional 20.7% (n= 3,106) of the participants declined to follow-up. This resulted in 77.6% (n= 11,727) who were eligible for the second wave (Table 1). To ensure that the sample remains representative of the wider population, the sample was refreshed by including an additional 1,142 new age-eligible participants (Table 1). Among these new participants 2.3% (n= 26) were proxy respondents, and 0.8% (n= 9) required LARs.

## MEASURES

The survey was catalogued into 10 sections (Table 2), with a broad range of topics related to physical and mental health, employment and retirement characteristics, financial status, utilization of healthcare and insurance, lifestyles and recreation, and cognitive function. These reflect psychosocial, socioeconomic, and health characteristics of the aging population in Singapore. Health related developments in the RHS assessed for cancer, high blood pressure, hypertension, cholesterol levels, diabetes, arthritis, depression, cognitive function, and dementia. Among these, diabetes, hypercholesterolemia, hypertension, dementia, and depression were identified as the top five chronic non-communicable diseases in Singapore [38].

Table 2 provides a brief overview and describes items measured in each section. To maintain consistency and comparability of data, efforts were made to ensure that differences between the survey items for the first and second wave were kept to a minimal. However, some changes were required to enhance the clarity and relevance of survey respondents. This primarily served to accommodate new priorities by the government in policy development.

Data collection was outsourced to a global survey company through a public tender process—was done via face-to-face interviews lasting between 1.5 hours to 2.0 hours. Participants were briefed about the scope and aims of the study, and informed that all responses were strictly confidential to maintain anonymity. The survey included questions about the respondents’ spouse or partner, participants were allowed to disclose information on their behalf or have their partners answer directly for those portions of the survey. Participants were contacted within two months of the interview if necessary to ensure accuracy of the information. Respondents were compensated for taking part in the survey with cash vouchers worth S$50 (circa US$37) if they completed the first wave of interview, and cash voucher of S$10 (circa US$7.40) upon completing the second wave of interviews.

To reinforce data representativeness, weights were assigned to all sample units indicating how the respective population sizes are represented by each unit, with adjustments for non-responses. Both longitudinal and cross-sectional weights can be applied to account for within sample and time variant characteristics. This ensured that samples were nationally representative of census for gender, ethnicity, marital status, education, and SES in Singapore. Although no physical examination was conducted during the interview process, the study was validated by forming limited linkages to relevant administrative data of the respondents, and this also circumvented missing data. The RHS sought approval from respondents to access administrative data from the MOH. This enabled cross-referencing health data such as mortality, morbidity, comorbidities, and disability. Additionally, this link gave information about public healthcare utilization such as usage of community clinics, out-patient visits, and medical expenditure. Linkage consent rate was 94% for wave one and 96% for wave two.

## KEY FINDINGS AND PUBLICATIONS

The RHS was primarily set up to track retirement and health trends, but this extends into evidenced-informed policy agenda setting, formulation, scenario planning, communication, and evaluation across domains of social and family development. Table 3 present a preliminary snapshot of demographics known to be important toward the relationship between retirement and health (age, race, citizenship, marital status, education, housing, and income). The data included adults from 45 years to enable researchers and policy makers to track retirement trajectories and transitions. The proportion of ethnicities were also representative of the overall ethnic composition in Singapore with a Chinese majority [39,40]. Broadly, the RHS include participants from low and middle SES, which is generally representative of our broader society in general. A large proportion of the population reside in mid-range priced public housing subsidized by Singapore’s HDB compared to more expensive private housing [39]. Also, a smaller number attained a higher education (i.e., post-secondary, degree, etc.), which is reflective of the characteristics of local baby boomers in the early 1950s [41].

We present a preview of health and retirement descriptors over two waves (Table 4). For comorbidities, high cholesterol and high blood pressure are the most prominent clinical diseases followed by diabetes among adults. Impairment for activities of daily living (ADL) were relatively consistent across most activities, but difficulties were relatively higher for managing finances and indoor mobility. A unique feature of the RHS was that it accounted for the intention to seek work as well as employment status. While there are studies that examine the intersection between work and wellbeing, few articulate how underlying motivations to engage employment impact on health outcomes among retirees [42]. In this data, most participants were either still under employment, or unemployed with no desire to seek employment (Table 4).

A report from the Central Providence Fund in 2019 from RHS data found that 42% of participants did not withdraw their pension funds, taking advantage of the interest rates from their pension scheme. Up to 51% of those who withdrew from their pension deposited the monies into private saving accounts and finance companies, reflecting a desire for liquidity among older cohorts. Decisions to withdraw or retain finances in their pension depend on the trade-off between meeting their current financial needs and investing their pensions funds for retirement. In the same report, 86% of working participants opted to transit into partial retirement before full retirement. Amongst them, 58% preferred to enter partial retirement by gradually reducing their work hours, and 33% preferred to reduce their workload. Beyond this, other fields of disciplines such as health and epidemiological research have used data from the RHS. Table 5 presents a summary of research papers that has used the RHS.

Two studies expanded on ADL disabilities with latent class analyses. The results indicate that instrumental activities of daily living (IADL) distinguished two classes of disabilities. In essence, difficulties with IADL were more salient for community dwellers compared to basic ADL. Furthermore, the studies demonstrated that disability is a dynamic process, with an approximately 40% likelihood of recovery from a disability. Conceptually, prevalence of recovery contributes to the understanding of physical resilience among older adults. The clinical significance of these studies highlights the need for community outpatient services to target rehabilitative efforts for more complex behaviors outlined by IADLs.

Another study examined the association between retirement intentions and employment after the statutory age of 67 in Singapore, where older adults with higher SES had a decreased risk of unemployment, while those from the manufacturing sector posed an increased risk of unemployment. This suggests that health care services need to help older adults clarify their intentions and attitudes toward retirement to prepare for productive aging. Additionally, two studies demonstrated that the lack of intention to seek employment increased the risk of developing disability over a two-year period, and inversely those actively looking for work demonstrated a higher probability of recovering from a disability. Engaging in meaningful work activities potentially help adults with disabilities recover toward independence. This meant that therapeutic efforts for disabilities may consider the benefits of purposeful engagement toward employment.

Beyond retirement and health, the RHS also found collaborations with other fields of study, exemplifying its contribution to a broader extent of the literature. Recently, a published study utilized the RHS to project long-term care needs. The researchers modelled not only functional disability, but also included social factors such as isolation and living arrangements [15]. Including social factors such as these to project long-term care has received less attention, especially for ethnic minorities in Southeast Asian societies [43,44]. They found that physical disability was projected to increase by five-fold, and social isolation would escalate by four-fold over the next 40 years [15]. The study also found ethnic disparities in social functioning, where Malays were more likely to be socially isolated compared to Chinese after adjusting for demographic variables [15]. Therefore, social functioning and ethnicity are potential factors to consider for local long-term care policies.

## STRENGTHS AND WEAKNESSES

There are several strengths and weaknesses. It is the first and largest nationally representative longitudinal study of aging in Singapore, providing significant detail on health, psychosocial, socioeconomic processes of aging. The sample is nationally representative with weights that closely approximate the socioeconomic and ethnic composition of Singapore’s population. This therefore provide valuable insights for evidence-based policy interventions at a national-level. The study’s biennial design ensures that longer term within-subject effects can be accounted for during modelling, with unbiased estimates of factors affecting health outcomes across the study population. Furthermore, the interview questions were carefully designed to provide rich resource for researchers undertaking research into the social wellbeing of seniors in Singapore. For instance, the survey design introduced an innovative focus on family structure. Finally, efforts were made to validate the survey by linking the respondent’s administrative data. This serves to cross-reference responses to ensure a high quality of reliability and representativeness of the data.

However, the RHS is not without limitations. Administering the questionnaire with a plethora of topics proved challenging. As such, it is difficult to strike a balance between the breadth of the survey and its ability to track aging factors in greater detail. The experience of aging can vary over time, and thus the importance of different aspects of lives can change as a function of social and environmental pressures [45,46]. Furthermore, the survey may need to consider that cohorts are not always homogenous, and aging can be experience differently according to the individual’s lifetime experiences [45,47]. This means that standardized procedures to alter items and procedures in the survey to fit the changing socioeconomic landscape of aging need to be articulated. Although best efforts were taken to validate responses via linkages with the respondent’s administrative data, the survey potentially remains open to a degree of self-report bias for psychosocial questions (e.g., social connectedness) for which there are no equivalent administrative data.

## Figures and Tables

**Table 1.** Details of deaths, dropouts, new participants, legally acceptable representatives (LAR), and proxy respondents over two waves of Singapore’s Retirement and Health Study

Variables	Wave 1 (n=15,013)	Wave 2 (n=12,869)
Proxy respondents^1^	314 (2.1)	151 (1.2)
Decline to follow-up^2^	3,106 (20.7)	-
Deaths	270 (1.8)	12 (0.1)
LAR	120 (0.8)	73 (0.6)
New participants	-	1,142 (8.9)

Values are presented as number (%).

1 Proxy respondents include spouses, partners, or children of respondents unable to carry out the interview.

2 Indicates the number of respondents who declined after completion of wave 1 to be followed up in wave 2.

**Table 2.** Overview of 10 sections in Singapore’s Retirement and Health Study

Section	Contents
Section A: Demographics	Basic demographic details such as age, gender, education, race, marital status of the respondent, household members, spouse/partner, and children
Section B: Physical, mental health, pre-existing conditions	Anthropometric measures, activities of daily living, health conditions, preventive care, depression, care-receiving and giving characteristics
Section C: Employment status, history and retirement	Respondents’ employment status and characteristics, job history, workplace features, and employment characteristics of spouse/partner
Section D: Financial background and status	Comprehensive assessment of assets, primary sources of income, residential property characteristics, attitude toward housing, debt, loans, liabilities, bequest, investments, life insurance, and includes financial information of spouse or partner
Section E: Sources of financial support and subsidies	Other sources include financial assistance from spouse, children, agency subsidies, family, ad-hoc and regular transfer of funds
Section F: Household expenditure	Amount spent on food, transport, recreation, utility bills, rent
Section G: Health insurance plans	Covers government schemes (e.g., Medishield, Eldershield), company healthcare benefit plans, family insurance, and any other insurance plans
Section H: Healthcare utilization	Consist of dental care, out/in-patient care, nursing costs, local/overseas surgery, home care services, day care usage, family healthcare expenses, usage of health aids, health supplements, and alternative treatments
Section I: Lifestyle factors linked to health	Assess for physical activity, smoking, drinking, social connectedness, and recreational lifestyles
Section J: Cognitive function	Brief screening of cognitive function including orientation, memory, and reading

**Table 3.** Demographic distributions of the cohort across two waves

Characteristics	Wave 1	Wave 2
Age (yr)		
45-49	2,465 (16.3)	2,096 (16.3)
50-54	2,718 (18.0)	2,320 (18.0)
55-59	2,450 (16.2)	2,085 (16.2)
60-64	2,226 (14.7)	1,966 (15.3)
65-69	1,861 (12.3)	1,620 (12.6)
70-74	1,597 (10.6)	1,386 (10.8)
75-79	1,083 (7.2)	884 (6.9)
80-85	703 (4.7)	512 (4.0)
Gender		
Men	7,861 (52.0)	6,691 (52.0)
Women	7,242 (48.0)	6,178 (48.0)
Ethnicity		
Chinese	8,073 (53.0)	6,843 (53.2)
Indian	2,515 (16.7)	2,223 (17.3)
Malay	3,455 (22.9)	2,875 (22.3)
Others	1,060 (7.0)	928 (7.2)
Citizenship		
Permanent resident	1,422 (9.4)	1,202 (9.3)
Singapore citizen	13,681 (90.6)	11,667 (90.7)
Marriage		
Divorced	851 (5.6)	809 (6.3)
Cohabitation	44 (0.3)	28 (0.2)
Married	11,090 (73.4)	9,241 (71.8)
Separated	90 (0.6)	72 (0.6)
Single	1,253 (8.3)	1,036 (8.1)
Widowed	1,775 (11.8)	1,683 (13.1)
Highest education qualification		
No formal education	3,089 (20.5)	2,667 (20.7)
Elementary school	2,724 (18.0)	3,127 (24.3)
Middle school	5,631 (37.3)	3,917 (30.4)
High school	786 (5.2)	696 (5.4)
Vocational school	464 (3.1)	418 (3.2)
Community college diploma	628 (4.2)	524 (4.7)
Degree and above	1,738 (11.5)	1,512 (11.7)
Missing/Invalid	43 (0.3)	8 (0.1)
No. of children		
1	2,040 (13.5)	1,739 (13.5)
2	4,797 (31.8)	4,031 (31.3)
>2	6,241 (41.3)	5,407 (42.0)
Missing/Invalid	2,025 (13.4)	1,692 (13.1)
Housing type		
Public 1-2-room flats	1,041 (6.9)	1,078 (8.4)
Public 3-room flats	3,363 (22.3)	2,821 (21.9)
Public 4-room flats	5,294 (35.1)	4,461 (34.7)
Public 5-room flats	3,621 (24.0)	2,975 (23.1)
Private condominiums	1,039 (6.9)	912 (7.1)
Private landed properties	745 (4.9)	621 (4.8)
Missing/Invalid	0 (0.0)	1 (0.0)
Residency		
Overseas	137 (0.9)	112 (0.9)
Singapore	14,965 (99.1)	12,757 (99.1)
Missing/Invalid	1 (0.0)	0 (0.0)
Household size (n)		
1	1,188 (7.9)	1,143 (8.9)
2-4	9,762 (64.6)	8,475 (65.9)
≥5	4,153 (27.5)	3,251 (25.3)
Household income		
Below median	12,499 (82.8)	10,561 (82.1)
Above median	2,604 (17.2)	2,308 (17.9)

Values are presented as number (%).

**Table 4.** Presents the proportion of physical diseases and retirement statistics

Variables	Wave 1	Wave 2
Physical and mental health		
High cholesterol	44.3	48.1
Diabetes	21.6	23.5
High blood pressure	43.4	46.4
Cancer	4.2	4.6
Dementia	1.2	1.2
Depression	3.5	2.7
Mobility (ADL)		
Mobility indoors	4.0	4.3
Mobility rooms	2.3	2.7
Move between bed	1.6	1.3
Self-care (ADL)		
Bathing	2.0	1.9
Clothing	2.0	1.7
Eating	1.4	1.0
Toilet	1.5	1.5
Instrumental ADL		
Telephone	1.1	1.1
Medications	1.7	1.7
Finance	2.1	2.1
Employment		
Fully employed	57.2	54.9
Unemployed seeking employment	3.8	3.3
Unemployed not seeking employment	38.7	41.8
Pursuing other activities (education, sabbatical, etc.)	0.3	0.0

Values are presented as percentage. ADL, activities of daily living.

**Table 5.** Sample projects that utilized data from the Retirement and Health Study

Title	Summary	Policy intention
40-Year Projections of Disability and Social Isolation of Older Adults for Long-Range Policy Planning in Singapore	Prevalence of disability is expected to grow five-fold over the next 40 yr, and socially isolated older adults is expected to grow five-fold	Policy agenda setting and capacity planning for long-term care
Retirement Attitudes and Employment Beyond Retirement	Older workers from the manufacturing sector show an increased risk of unemployment beyond retirement	Targeted implementation of government matching programs for re-employment of older workers nearing retirement age
Resilience from Disability among Older Adults	Psychosocial factors such as the motivation to work are linked to recovery transitions from disability	This project laid the groundwork to design resilience programs for older adults to promote recovery from disabilities
Frequent Admission to Public Hospitals	This project sought to distil the psychosocial factors associated with frequent admission to public hospitals in Singapore	Insights from this study will lay the groundwork to design social service and community programs to decrease the risk of frequent readmissions
